# A randomized controlled trial comparing Mindfulness and Heartfulness meditation programmes on stress, anxiety, depressive symptoms and wellbeing among distance-learning postgraduates

**DOI:** 10.3389/fpsyg.2026.1810901

**Published:** 2026-09-17

**Authors:** Diana Brito Pearmain, Mark Kennedy, Karoly Schlosser

**Affiliations:** 1 Institute of Psychiatry, Psychology and Neuroscience, King’s College London, London, United Kingdom; 2 Department of Child and Adolescent Psychiatry, Institute of Psychiatry, Psychology and Neuroscience, King’s College London, London, United Kingdom

**Keywords:** anxiety, depression, Heartfulness, Mindfulness, postgraduate students, remote intervention, stress, wellbeing

## Abstract

**Background:**

Postgraduate students are six times more susceptible to mental health challenges than the general adult population, making it imperative to identify effective initiatives to support them. This is particularly relevant for master’s and doctorate students undertaking distance-learning programmes as this is a dimension of the student experience that remains unexplored. Meditation practices such as those included in Mindfulness and Heartfulness programmes have been shown to improve university students’ mental health and wellbeing even when delivered via videoconferencing. However, the efficacy of these approaches remains under-investigated among distance learning postgraduates.

**Objectives:**

This study aims to explore the impact of an online synchronous Mindfulness-based intervention (MBI) compared to a Heartfulness-based intervention (HBI) on distance-learning postgraduate students’ mental health and wellbeing using randomised controlled trial (RCT) design.

**Methods:**

The MBI included guided meditations, emphasizing present moment focus through breathwork and body scan exercises, compassion and non-judgmental observations. The HBI included a heart-focused form of silent meditation, with additional mental exercises aimed to facilitate the clearing and release of impressions. Weekly live online instructor-led group sessions facilitated access across 17 countries on six continents. In an initially powered RCT using block randomisation, *N* = 73 UK-based and international postgraduate students from King’s College London (KCL) were allocated to either HBI (*n* = 37) or MBI (*n* = 36). 61 (80% female) completed validated questionnaires at baseline (T1), after 8-week intervention (T2) and at twelve-week follow-up (T3) through intention-to-treat (ITT) analysis. Significance was assessed through repeated-measures ANOVA, supplemented by mixed-effects modeling to assess robustness.

**Results:**

In line with hypotheses, the group × time interaction was not statistically significant for primary outcomes, providing no indication of differential change between interventions. Both MBI and HBI were associated with significant improvements in stress, anxiety, depressive symptoms and wellbeing in postgraduates over time, established through secondary within-group outcomes at T2. At T3, improvements were maintained for wellbeing and stress through ITT analysis. Anxiety reductions were sustained only through Per-Protocol (PP) analysis at T3.

**Conclusion:**

These findings are consistent with comparable effects. Both HBI and MBI may be beneficial as preventative and transdiagnostic approaches for postgraduate students’ mental health and wellbeing, at scale, and remotely with a global reach.

## 1 Introduction

Student mental health is an increasing challenge worldwide within higher education institutions and schools (Storrie et al., 2010; Lee, 2021; Feiss et al., 2019). Postgraduate students consistently rank at the highest risk of acquiring elevated depression symptoms, anxiety, and stress (DAS), or burnout during their academic journey, compared with the general adult population and undergraduate students (SenthilKumar et al., 2023; Chi et al., 2023; Brooke et al., 2020; Metcalfe et al., 2018). Evidence across 26 countries suggests they are over six times more susceptible to experience anxiety and depression (39% versus 6%), (Evans et al., 2018; Kocalevent et al., 2013). Their risk of mental health issues has been persistently reported (Guo et al., 2021). These findings are concerning because the symptoms can affect both academic performance and long-term wellbeing. They have been linked to up to 50% of postgraduate students’ main causes for their decisions to either pause or leave academia (SenthilKumar et al., 2023).

In the past 5 years since the COVID-19 pandemic in 2020, there has been a substantial increase in remote postgraduate programmes, as reported by the Higher Education Statistics Agency. There are up to 65% more enrolments amongst all UK-based postgraduates (from 17% in 2018/2019 to 28% in 2023/2024), a total of 93,900 new online admissions (Mosley, 2025). This shift is likely to sustain. The mental health challenges faced by remote learning students, however, remain underexplored in research literature (Chung et al., 2021). For this reason, strategic emphasis on this target group with potentially implementable mental health support programmes is imperative.

Randomized Controlled Trials (RCTs) that focus on postgraduate students’ mental health and their DAS’ levels remain limited, especially in the context of international distance learning programmes. An 8-week synchronous online Mindfulness-based intervention (MBI), a brief version of the Mindfulness-based Cognitive Therapy (MBCT), showed evidence of significant reduction in “emotional disturbances” (*p* = 0.015), enhancement of resilience (*p* = 0.012), wellbeing literacy (*p* = 0.04) and trait mindfulness (*p* = 0.006) among postgraduates in Hong Kong and Macau (Xu et al., 2025). Recent meta-analysis also revealed that online MBIs significantly decrease stress (95% confidence interval [CI], −0.79 to −0.37; *p* < 0.00001), anxiety (95% CI, −080 to −0.14; *p* = 0.006) and depression symptoms (−0.48 to −0.07; *p* = 0.008) in university students (Gong et al., 2023).

Despite the limited trials published about Heartfulness, a recent article released in 2024 highlighted that a daily 12-week synchronous web-based Heartfulness programme RCT with 78 Health Care students presented significant reduction on stress (*p* < 0.001), anxiety (*p* = 0.006) and depressive symptoms (*p* = 0.02) (Thimmapuram et al., 2024). There are currently no existing studies focused on Heartfulness Meditation supporting postgraduates or being compared with any active condition in the scientific literature. There is also limited research analyzing DAS prevalence in master’s/taught students explicitly, either on campus or remotely (Coneyworth et al., 2020; Miles, 2024).

### 1.1 Prevalence of DAS among the postgraduate student population

A meta-analysis of 39,668 postgraduate students investigated worldwide showed that more than 1 in 4 master’s students experience anxiety symptoms (29.2%) (Chi et al., 2023). This research has shown an upward trend of this prevalence since 2005, with the incidence even higher among doctoral or PhD students (34.3%), with 1 in 3 suffering from anxiety (Chi et al., 2023). Studies also evidenced women face increased risk of mental health disorders compared with men, with the transgender and gender-diverse populations also more likely to suffer from anxiety and depression (Eaton et al., 2012; Dhejne et al., 2016; Evans et al., 2018). Postgraduates were also significantly impacted during the COVID-19 pandemic by stress and psychological health issues (Andrade et al., 2023; Corrêa et al., 2022; Aubin et al., 2025). Limited integration or social isolation, feelings of loneliness, financial strain and living conditions, such as being away from support networks, are among the possible contributors of DAS, which is evidenced to be as high as 43% amongst the UK international postgraduate population (Hosseinpur et al., 2023; Ejim et al., 2021). This evidence demonstrates that globally universities face the challenge of providing effective support at scale, not just on-campus but in remote study conditions. Mental health support initiatives may mitigate symptom escalation and lower the risk of chronic mental health conditions (Guo et al., 2021).

### 1.2 Demographic variables, stress, and isolation as transdiagnostic risk factors of mental illness

Stress appears to be a transdiagnostic risk factor, and possibly perpetuating determinant, for developing various physical and mental health conditions among the general population (Remmerswaal et al., 2024; Liu et al., 2021; Schlosser, 2024a; Ernst, 2025). These include cardiovascular disorders, cancer, stroke, muscular and chronic pain disorders, as well as anxiety and depression, which are leading causes of economic and social burdens. Of these, as per the World Health Organization (WHO)’s projections, depression could become the global top major cause of disease by 2030 (WHO Executive Board, 2012; Trautmann et al., 2016). Indeed, since the COVID-19 pandemic, there has been a steepening increase observed in mental health concerns, with depression rising 27.6%, and anxiety 25.6% (COVID-19 Mental Disorders Collaborators, 2021). Findings show that demographic factors, such as age, sex, and socioeconomic status were significant determinants of mental health conditions during the pandemic: being female, an adolescent or young adult, people in regions experiencing greater levels of loneliness and stress during lockdowns, or being from lower or mid-level income regions (COVID-19 Mental Disorders Collaborators, 2021).

### 1.3 Critical need to support distance learning postgraduate students’ mental health

Despite growing discussion about a mental health crisis among postgraduate students (Evans et al., 2018), preventative mental wellbeing programmes addressing the distance learning postgraduate population remain rare (Berry et al., 2021). Therefore, it has become imperative to explore alternative and/or complementary approaches (Chung et al., 2021) to the traditional mental health support system offered by universities, such as counseling, as recommended by regulatory bodies, including the Office for Students (OfS) in the UK. Despite persistent efforts, there remains a shortfall in students’ accessibility to mental health services. Reasons cited may be due to students not having sufficient information on services available, as well as stigma associated with accessing support, and limited culturally informed mental-health assistance for students from diverse backgrounds (Priestley et al., 2022; SenthilKumar et al., 2023). Support programmes that are available often offer asynchronous digital self-administered large-scale approaches (meetings that are not taking place in real time). Although these are at low cost, they may not facilitate interaction with peers and trainers (Galante et al., 2018; Roxburgh, 2022), which may be necessary to decrease the sense of isolation and loneliness. Individuals studying in isolation can form friendship and community when using a synchronous online platform (Nicholson, 2002; Oztok et al., 2013; Hrastinski, 2006). In real time (synchronous) interventions, the teacher or instructor is also available to address unwanted experiences directly and can offer one-to-one support, helping students in any potential crisis (Van Dam et al., 2025).

### 1.4 Mindfulness-based interventions (MBIs) and the mental wellbeing of students

Mindfulness is the practice and state of bringing awareness to the present moment using focus, acceptance and non-judgment (Kabat-Zinn, 1990). This can be developed into a skill that is applied to tackle transdiagnostic challenges in everyday life (Antonova et al., 2021). MBIs are psychological and psychotherapeutic approaches that use mindfulness exercises aiming at stress-reduction and at improving mental health (Lin et al., 2022), through practicing techniques of focused breathing, body scan, mindful exercises, and cultivating compassion. These exercises originate from Buddhism practices of the Vipassana and the Zen tradition (Kabat-Zinn, 2003) and were developed into a secular form of mindfulness practice called Mindfulness-based Stress Reduction (MBSR) (Kabat-Zinn, 1990) to support of patients living with chronic illness by reducing the associated pain and stress. The MBSR programme has also been evidenced to reduce the functional activity of the amygdala to enhance its neural connectivity with prefrontal cortex and the hippocampus, a complex brain structure linked to regulation of emotions in response to stress, anxiety and fear (Kabat-Zinn, 2003; Hölzel et al., 2010). Its subsequent alteration termed Mindfulness-based Cognitive Therapy (MBCT) was developed for preventative treatment of depression relapse (Kuyken et al., 2008), focusing more on cognitive-behavioral aspects. Smaller-scale preventative programmes often show more benefits than larger and compulsory trials such as the My Resilience in Adolescence (MYRIAD) project. This offered Mindfulness training within school time but had lower-than-expected engagement, with concerns arising related to relatively inexperienced mindfulness instructors with limited training (Kuyken et al., 2022; Strohmaier and Bailey, 2023). In addition, a meta-analysis of MBIs revealed that there are limited experimental head-to-head trials investigating the efficacy and comparison of MBIs with active conditions for prevention of mental-health challenges, with most focusing on inactive groups (Ma et al., 2019; Liu et al., 2024; Galante et al., 2021).

### 1.5 Heartfulness as a heart-focused meditation approach to support students’ mental wellbeing

The Heartfulness practice is based on the classical system of Raja Yoga (Sahaj Marg meaning “the natural path”), focusing on a heart-centered meditation (Van’t Westeinde and Patel, 2022). The method was founded in 1940 by Ram Chandra, known as Babuji Maharaj, whilst the current contemporary form of Heartfulness meditation was introduced by Kamlesh Patel in 2015 for empirical appraisal (Thimmapuram et al., 2017; Patel and Pollock, 2018). The practice can often involve a brief body scan relaxation prior to setting an initial intention to direct awareness toward an assumption of a subtle source of light within the heart, also described as the “inner self” (Van’t Westeinde and Patel, 2022). Unlike Mindfulness, which offers meditation with spoken guidance in the background, Heartfulness focuses on deep silent meditation. It is described as a meditation that employs guidance from a trainer involving a technique called Pranahuti or “yogic Transmission,” also known as original source or essence, suggested to act directly on the heart (Patel and Pollock, 2018). Heartfulness aims at self-awareness and inner balance (Patel and Pollock, 2018; Van’t Westeinde and Patel, 2022). An additional mental exercise implemented as part of the practice is proposed to facilitate the clearing and release of impressions or patterns to support emotional-cognitive regulation (Telles et al., 2023; Naseem and Khalid, 2010). Aligned with evidence provided in a meta-analysis showing that MBIs can lead to changes in parts of the brain linked to mood regulation (Siew and Yu, 2023), a recent study (Dessain et al., 2023) suggests Heartfulness meditators with years of practice may enhance mood control presenting an increased structural connectivity of the hippocampus, especially in the right posterior area, which could be predicted by Diffusion Magnetic Resonance Imaging (dMRI) data. The practice may also decrease feelings of burnout, stress and loneliness, improving sleep and wellbeing (Thimmapuram et al., 2017; Iyer et al., 2023; Desai et al., 2021). For over 10 years, the National Institute for Health and Care Excellence (NICE) has recommended Mindfulness-based therapies to treat mild to moderate depression in the UK through the National Health Service (NHS). Since 2022, NICE has also proposed Mindfulness, yoga or meditation (in general), as alternatives to support mental health and wellbeing in the workplace.

### 1.6 The current study

This is the first experimental evidence focusing specifically on the mental health of distance learning postgraduate students, who are more likely to be isolated without the same level of access to initiatives as on-campus students. This initially powered head-to-head RCT aims to compare the equivalence and effectiveness of a Heartfulness-based intervention (HBI), with a Mindfulness-based intervention (MBI), in reducing perceived stress, anxiety and depressive symptoms and in enhancing wellbeing, among distance master’s students. The current research aims to address the following questions: (1) How do the HBI and MBI affect distance-learning postgraduate students’ wellbeing, stress, anxiety and depressive symptoms? (2) Did each intervention enhance outcomes from baseline? (3) Did MBI or HBI differ from each other in their effects on the outcomes? The following hypotheses was proposed: We expect both MBI and HBI to deliver equivalent and significant improvements in wellbeing, stress, anxiety and depressive symptoms following the interventions, and that these positive changes would persist at follow-up.

## 2 Materials and methods

### 2.1 Design

An exploratory, initially powered head-to-head Randomised Controlled Trial (RCT) was conducted virtually between August and December of 2024 to investigate and compare the effects of an 8-week MBI or HBI in elevating wellbeing and reducing depressive symptoms, anxiety and stress (DAS). The study is considered a mixed design, combining both between-subjects factors (MBI compared to HBI), and within-subjects (time at three points: baseline (T1), 8-week post-intervention (T2), and at 12-weeks follow-up (T3). Primary analyses focused on between-group differences across psychological outcome measures, while within-group pre-post changes were treated as secondary analyses.

Block randomisation (McEntegart, 2014) was employed to maintain balanced allocation between intervention arms (MBI and HBI) during enrolment. Participants were recruited from similar distance-learning settings. Although participants were recruited internationally from two distance-learning master’s programmes from the Institute of Psychiatry, Psychology and Neuroscience (IoPPN) at King’s College London (KCL), in the UK, randomisation was not stratified or restricted by demographic variables such as gender, age, or nationality.

### 2.2 Participants and eligibility criteria

Study inclusion criteria determined that participants must have been enrolled in the 2-year distance master’s programmes of Psychology & Neuroscience of Mental Health (PNoMH) Master of Science (MSc) and Applied Neuroscience MSc from the IoPPN at KCL, in the UK. Exclusion criteria for being admitted into the intervention groups included current self-reported use of psychiatric medications to support their mental health that could affect autonomic, neurological or mood systems such as hological arousal moderating effectiveness of meditation under inveantidepressants and antipsychotics. This was because such medications may alter focus, affects or psycstigation, which could confound or impact the outcomes (Hoge et al., 2023; Andreu et al., 2019). The Psychology and Neuroscience of Mental Health distance master’s programme team facilitated recruitment. A flyer advertising the study was developed by one the authors of the study and announced via email to the targeted distance master’s students by the programme team, including the exclusion criteria.

Students had 2 weeks to confirm participation through a signed consent form and Assessment Form, alongside completion of baseline questionnaires and Participant Information Sheet, indicating participation would be voluntary. The students also received an incentive from King’s College London, with 10 vouchers of 50 pounds randomly allocated to those completing the intervention. Following completion of baseline self-assessments via Qualtrics, students were randomly assigned to the treatment groups.

### 2.3 Sample size and procedures

The sample size was estimated using G*Power 3.1 (Faul et al., 2009) for a mixed repeated measures ANOVA (2 groups × 3 time points), assuming a medium effect size (Cohen’s f = 0.25) (Cohen, 1988), a = 0.05, and power = 0.80. This yielded an estimated required analysable sample of approximately *N* = 80 participants. To account for anticipated attrition loss (25%) to interventions expected due to time and effort required for participation, the target recruitment sample was increased to 106 participants (53 per group). The mean age in the Mindfulness group was 35–44 years old (40%) and in the Heartfulness cohort was 45–54 years old (35.5%). While in the Mindfulness group most of participants were employed full-time (33%) and already meditated sometimes (13.8%), in the Heartfulness group 35.5% were self-employed and 19.4% also practiced meditation occasionally. Participants from both intervention groups reported a wide variety of national identities: British (16.4%), Chinese (16.4%) were most prevalent, with 64% from somewhere else around the world. Just over a quarter (26%) were residents of the UK.

Overall, 90 students indicated their interest in the study, with 73 participants then randomized, 61 (80% female) completed validated questionnaires at baseline (T1), post-intervention (T2) and post-follow-up (T3) through intention-to-treat (ITT) analysis, with, respectively 30 in the Mindfulness cohort (83% female), and 31 in the Heartfulness group (77% females), as shown in the Figure 1. A total of 57 completed the intervention through per-protocol (PP) analysis. The achieved sample size was slightly lower than the planned target and therefore may have reduced sensitivity to detect moderate between-group effects. However, the achieved sample size remains comparable to many MBI RCTs reported in previous meta-analytic literature (Hofmann et al., 2010) which were considered moderate in terms of effect size, with an average of 34–60 participants per study. Meta-analytic evidence specific to Heartfulness meditation remains limited, so sample size estimation was based on effect sizes reported in related MBIs to align with the comparable effects hypothesis.

**FIGURE 1:**
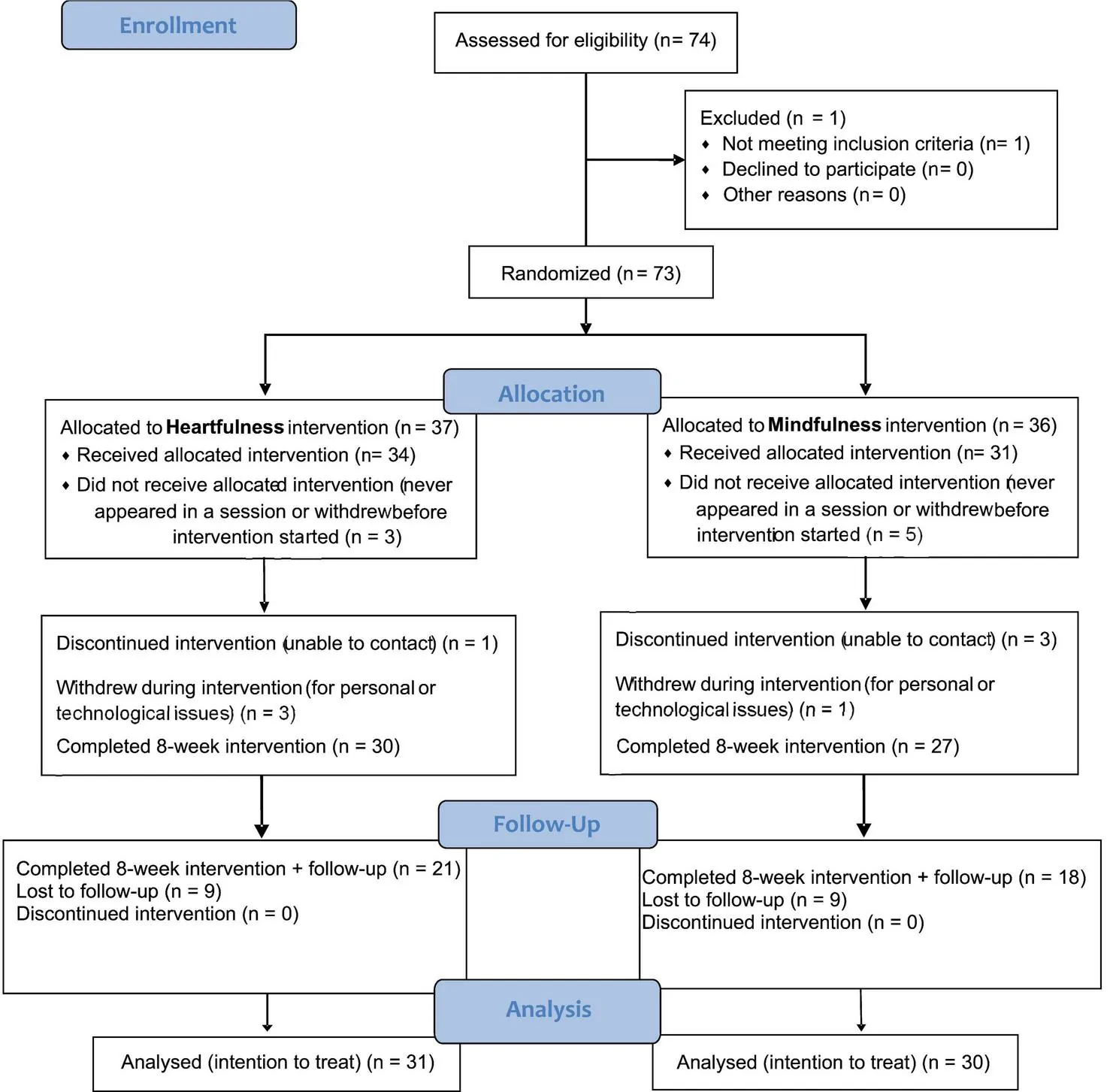
Participants flow diagram. Consolidated Standards of Reporting Trials (CONSORT) diagram: flow of participants.

### 2.4 Interventions

Both MBI and HBI interventions were delivered as a brief synchronous online guided programme over an 8-week period, with additional monthly recap sessions over the 12-week following the eight-week period completion. The whole programme was delivered in a total of five months, from August to December 2024. However, to be the same length as the HBI condition, the MBI was trimmed to 1 h per week length exposure. Below, under the interventions section, the framework used for the development of the MBI, as an abbreviated Mindfulness condition, is outlined.

Group-based Mindfulness and Heartfulness meditation sessions were offered via qualified instructors as part of a bespoke programme once a week for 8 weeks (1 h each session) in August and September 2024. Both the MBI and HBI had a group at noon and another cohort early evening to facilitate access for participants to attend training when dialing in from worldwide locations in 17 countries across six continents. Follow-up sessions also took place once a month (twice a day at noon and early evening) for each of the three scheduled months in a similar manner.

Two MBI and two HBI groups were carried out via live online instructor-led sessions once a week during each of the 8 weeks on Microsoft Teams (two groups on Mondays for Mindfulness at noon or early evening and two groups on Wednesdays for Heartfulness also at noon or early evening). The number of participants at noon and early evening for both MBI and HBI interventions were carefully distributed and balanced to avoid bias. Every MBI and HBI group-based session was supervised by one of the members of the research team to ensure consistency and fidelity to the planned programme.

#### 2.4.1 The mindfulness-based intervention (MBI)

The brief MBI was focused on expanding self-awareness based on the MBCT workbook. Participants allocated to the MBI group received live online instructor-led sessions for 1 h by an experienced teacher formally trained at the Oxford Mindfulness Centre (OMC) with nearly a decade teaching MBCT. The teacher also had a background in psychology and experience in Mindfulness practice within a clinical support context. The MBI included 20–50 min homework per week, which focused on reinforcing various elements of the practice. However, there was no formal record of adherence to the homework assignment.

During the sessions participants were encouraged to assess self-observation through focused breathing, body scan exercises, awareness and connection of mental events (such as automatic pilot), as well as to work with difficulty and to experience non-referential compassion. The sessions also directed participants to bring attention to a part of the body (present moment focus) to anchor through the senses. Breathing exercises, guided meditation linked to body-scan relaxation, acceptance, self-compassion and cultivating mindfulness practices to observe thoughts and feelings without judgment were also offered.

The regular MBCT training is 2.5 h per week, with daily homework of 30–60 min. To match the Heartfulness Intervention condition in length, the need to design a briefer MBCT-based intervention arose. This development was achieved through the use of the Functional-contextual Skill-acquisition Model, which was used as a framework to select experiential MBCT exercises that are effective to build and develop mindfulness as a trait and skill (Schlosser, 2018, 2024b; Schlosser et al., 2025). This approach aims to be responsive to individual needs for precision purposes responding to recent challenges identified in large scale interventions (Montero-Marin et al., 2022; Macías et al., 2022; Schlosser, 2024b). Integrating this approach was necessary to develop a brief version of MBCT that is effective to facilitate the formation of adaptive and resilient coping skills, and to foster mental health and wellbeing among participants in remote contexts, as well as potentially in crisis.

#### 2.4.2 The Heartfulness-based intervention (HBI)

The HBI was conducted weekly via a 1-h live online instructor-led session with two qualified Heartfulness instructors, who both had over a decade of experience, trained at the headquarters of the Heartfulness Institute (HI) in India, in Hyderabad, Telangana. The trainers also had a professional background in psychology, including one who has experience working in a clinical setting. Homework was also suggested for 20–50 min a week, which focused on specific components of the practice each week. There was no formal record of adherence to the homework assignment.

During every session, participants were initially introduced to parts of the Heartfulness system, then guided through a brief body-scan relaxation before completing group meditation in deep silence for 20–30 min. The trainer advised the participants to close their eyes focusing their attention on an assumption of a subtle source of light emerging from their hearts (Thimmapuram et al., 2024, 2017; Iyer et al., 2023) then relaxing into this awareness. The instructors encouraged participants to carefully refocus toward their hearts if they lost attention (Thimmapuram et al., 2024). After meditation, participants were asked to observe their experiences, write notes and share sensations with the group if they wished. An additional mental exercise was also presented as a guided process within the sessions aiming at clearing and release impressions and lingering emotions or pattens to support cognition and emotional balance (Van’t Westeinde and Patel, 2022). Participants were recommended to then introduce the elements of the approach routinely if they were able to.

### 2.5 Measures

This study used six well-established validated self-reporting questionnaires, which took participants an average 10 minutes at baseline, post-intervention, and follow-up.

#### 2.5.1 Perceived stress Scale-10 (PSS-10)

The shorter 10-item version of PSS was used to assess the degree of individuals’ perceived stress symptoms experienced in the last month (Cohen, 1988). Responses are presented by 5-point Likert scale (0 = never, 4 = very often). Total score ranges from 0 (low stress) to 40 (high stress). The PSS-10 scale demonstrated good internal consistency with a Cronbach’s alpha (α) of 0.87 at baseline and 0.84 post-intervention, confirming steady reliability. The internal consistency of the PPS-10 is often reported among 0.80 and 0.90 in many studies (Sun et al., 2019; Glasscock et al., 2018) suggesting it is a reliable scale for measuring perceived stress.

#### 2.5.2 The patient health questionnaire-9 (PHQ-9)

The 9-item PHQ version, measured frequency and severity of depressive symptoms within the past 2 weeks (Kroenke et al., 2001). The alternative responses are presented by 4-point Likert scale (0 = not at all, 3 = nearly every day). Total score ranges vary between 0 and 27, and elevated scores suggest severe depression. This scale showed good internal consistency in the current study (baseline α = 0.84, post-intervention α = 0.79), as many previous studies using this tool (Gilbody et al., 2007; Alreshidi, 2024) suggesting it is a reliable scale for measuring depressive symptoms.

#### 2.5.3 Generalized anxiety disorder scale-7 (GAD-7)

The 7-item GAD measured anxiety symptoms (Spitzer et al., 2006) between 0 and 21 points (elevated scores indicate higher severity). The alternative responses are presented by four-point Likert scale (0 = not at all, 3 = nearly every day) assessing the past 15 days. The scale shows good internal consistency in this study (baseline α = 0.90, post-intervention α = 0.85), as large-scale previous studies using this tool (Corpas et al., 2021; Richards et al., 2016) indicating its reliability.

#### 2.5.4 The world health organization-five wellbeing index (WHO-5 WBI)

The short 5-item WHO assessed general and subjective psychological wellbeing (positive mood, vitality and interest in life) over the last 2 weeks (World Health Organization, 1998). Each item scored on a six-point scale (0 = at no time, 5 = all of the time). Total score ranges vary between 0 and 25 and higher scores represent the best possible quality of life. This scale shows good internal validity in this study (baseline α = 0.82, post-intervention α = 0.88), as many previous studies using this tool (Coelhoso et al., 2019; Maatouk et al., 2018) representing WHO-5 as a reliable scale for measuring wellbeing levels.

#### 2.5.5 Short Warwick-Edinburgh mental wellbeing scale (SWEMWBS)

Aiming to capture the unidimensional nature of positive mental wellbeing, the seven-item SWEMWBS derived from this conceptual framework (functioning, personal growth and satisfying relationships) over the past 2 weeks (Tennant et al., 2007; Stewart-Brown et al., 2009). The responses are presented by five-point Likert scale (1 = none of the time, 5 = all of the time). Total score ranges vary between 7 and 35 and the higher scores indicate higher positive mental wellbeing. This scale shows good internal consistency in the current study (baseline α = 0.82, post-intervention α = 0.83), as other previous studies using this scale (Vaingankar et al., 2017; Ng Fat et al., 2017) indicating it is a reliable measure of wellbeing.

#### 2.5.6 Subjective happiness scale (SHS)

While the four-item SHS scale measured subjective self-report sense of happiness, which can be considered key component of wellbeing (Lyubomirsky and Lepper, 1999). The measure consists of four items on a seven-point scale (e.g., 1 = not a very happy person, 7 = a very happy person). The total range of scores is from 1 to 7 and the higher scores indicate greater happiness. The internal consistency for this study was excellent (baseline α = 0.92, post-intervention α = 0.91), indicating reliability of the scale. Good consistency was also reported in previous studies (Alquwez et al., 2021; Feliu-Soler et al., 2021).

### 2.6 Statistical analysis

The researchers chose the Repeated Measures Analysis of Variance (RM ANOVA) analytical approach because there are peer-reviewed articles (Bakeman, 2005; Algina and Olejnik, 2003) providing support for this test to be used for mixed design studies, suggesting it enhances statistical power and validity by reducing error variance and controlling for individual differences (Field, 2018). While a Paired T-test would compare only two time points for each participant, the RM ANOVA amplifies the within-subjects analysis considering all time points simultaneously (T1, T2, and T3) x group (MBI vs. HBI) and also testing between-subject (primary outcomes) effects and interactions (Bakeman, 2005; Field, 2018). Although the preregistration specified MANOVA, RM ANOVA was used as it better accommodated the repeated-measures structure of each outcome, incorporating both the between-group intervention factor (primary analyses) and within-subject changes over time. Supplementary mixed-effect modeling were performed to confirm robustness of the results.

The RM ANOVA was conducted separately for each of the six scales of the four primary outcome variables (stress, anxiety, depressive symptoms, and wellbeing), to investigate any positive intervention effect on the distance master’s students’ mental health in terms of reducing stress, anxiety, depressive symptoms and increasing wellbeing levels, respectively. The study was initially powered to detect intervention effects on stress, anxiety, depressive symptoms and wellbeing at the 8-week post-intervention assessment, which was defined as the primary endpoint.

All the data was analyzed using SPSS Statistics for Windows, Version 29 (IBM Corporation, Armonk, NY, USA). ITT was considered as the primary analysis of this study and included all randomized participants in their original assigned group, independent of adherence through last observation carried forward (LOCF) method. While LOCF may underestimate change, results were consistent with mixed-effects models, suggesting findings are robust to missing-data assumptions. Sensitivity analyses were conducted through the per-protocol (PP) analysis, with participants that completed the intervention (dependent on adherence) as assigned, indicating consistency of results to different missing-data assumptions. *P*-values < 0.05 indicate statistically significant results.

### 2.7 Ethical approval

The Helsinki Declaration of ethical principles were followed in this study (Ashcroft, 2008). This research was approved on 3 May 2024 by the Institutional Review Board or Ethics Committee of the Health Faculties at King’s College London (protocol no.: HR/DP-23/24-41111). The study was preregistered on the Open Science Framework (OSF) prior to data analysis (OSF Registries osf.io/48GFT; https://doi.org/10.17605/OSF.IO/48GFT).

Ethical principles were upheld following the British Psychological Society (2021) including informed consent, confidentiality, and participant welfare (participants had the right to withdraw from the study at any time). Each participant’s name was anonymized and substituted by an identifier pseudonym and number after transcribing the data, following the terms of UK data protection law, including the UK General Data Protection Regulation (UK GDPR), the Data Protection Act 2018 and the British Psychological Society (BPS) code of human research ethics (updated 2021).

The Participant Information Sheet (PIS) provided details about the interventions prior to the participants confirming their participation. A notification was included to signpost participants to mental health support (24h Samaritans service and KCL), if appropriate, based on the screenings conducted as part of the study, or in case of any future disclosure, as well as due to the relatively sensitive nature of questionnaires, for example PHQ-9 items about suicidal thoughts. There were no serious adverse events among participants during the trial. Considering the non-invasive and low-risk nature of the behavioral intervention, an independent Trial Steering Committee (TSC) or Data Safety Monitoring Board (DSMB) was not established. Participant safety was monitored by the research team and the institutional ethics committee in accordance with approved study procedures.

## 3 Results

### 3.1 Demographic characteristics of the study participants

Independent samples T-tests and Chi-square analyses showed no significant difference in age between MBI (*M* = 3.1, SD = 1.1) and HBI (*M* = 3.2, SD = 1.0), *F*_(1, 59)_ = 0.007, *p* = 0.93. Also, no significant differences were observed in gender, employment status, marital status, children, current wellbeing activity, national identity and country of residence between the interventions (all *p* > 0.05). This confirms that randomisation successfully created equivalent groups at baseline (T1) (see Table 1). A total of 73 participants were randomized to the MBI group (*n* = 36) and HBI cohort (*n* = 37). However, eight were excluded from analysis (five from the MBI, three from the HBI) due to lack of baseline data. They completed a Consent Form but did not complete questionnaires and did not respond to contacts. At T2, [8 (12.30%) of 65 participants (4 (12.90%) in the MBI group and 4 (11.76%) in the HBI cohort] discontinued participation. Some of the reasons for dropout or withdrawal were that participants had never appeared in a session and were unable to be contacted, or informed that they were busy attending to work, coursework deadlines, or personal matters. By T3, a further 18 participants {27.69% [9 (41.93%) in the MBI group, 9 (38.23%) in the HBI cohort]} were lost to follow-up without justification or because of work commitments, resulting in 57 participants (87.69%) completing all assessments after MBI (*n* = 27) or HBI (*n* = 30), and also 39 participants (60%) completing all assessments MBI (*n* = 18) and HBI (*n* = 21) post-follow-up. The overall attrition rate was 40% (41.93% for the MBI cohort, 38.23% for the HBI group). The missing scores were imputed using LOCF including all 65 participants that completed questionnaires at baseline (T1) through ITT analysis.

**TABLE 1:** Sample characteristics.

Distance master’s students from IoPPN	Mindfulness	Heartfulnes	Total (both groups)
	30 participants (%)	31 participants (%)	61 participants (%)
Age			
35–44	40	29	34.4
45–54	23.3	35.5	29.5
27–34	13.3	22.6	18
Over 55	13.3	9.7	11.5
19–26	10	3.2	6.6
Gender			
Female	83.3	77.4	80.3
Male	16.7	12.9	14.8
Non-binary/third gender	0	9.7	4.9
Employment status			
Self-employed	26.7	35.5	31.1
Employed full-time (40 h/week)	33.3	29	31.1
Unemployed student (looking for work)	20	12.9	16.4
Employed part-time (less 40 h/week)	10	12.9	11.5
Unemployed (not looking for work)	10	9.7	9.8
Marital status			
Single	36.7	32.3	34.4
Married	56.7	58.1	57.4
In a domestic partnership	6.7	6.5	6.6
Divorced	0%	3.2	1.6
Children			
Yes	50%	51.6	50.8
No	50%	48.4	49.2
Current wellbeing activity			
No and i never meditated before	10.3	12.9	11.7
I meditated before but i stopped	37.9	9.7	23.3
I am still meditating sometimes	13.8	19.4	16.7
Yes, i exercise at least twice a week	17.2	41.9	30
Other wellbeing activity	20.7	16.1	18.3
National identity			
UK	16.7	16.1	16.4
China	16.7	16.1	16.4
USA	6.7	0	3.3
Other (Portugal, Romania, Japan, Brazil, Canada, and others)	60	67.7	63.9
Country of residence			
UK	26.7	25.8	26.2
China	10	12.9	11.5
USA	6.7	9.7	8.2
Other (Portugal, Romania, Japan, Brazil, Canada, and others)	56.6	51.6	54.1

Demographic description of participants.

### 3.2 Between-group differences in MBI and HBI – primary outcomes

As presented in Table 2 the baselines mean scores for DAS and wellbeing did not initially differ meaningfully between the MBI and HBI groups. Between-group effect sizes (Cohen’s d) were all small (range: −0.28 to 0.07), showing that randomization successfully produced comparable groups prior to the interventions. The between-group differences were small, and 95% CIs crossed zero, indicating no statistically significant differences between HBI and MBA (group x time) for primary outcomes.

**TABLE 2:** Descriptive statistics.

Intension-to-treat analysis								
Outcome	Treatment group	T1/baseline (mean, SD)	T2/8 weeks post-intervention (mean, SD)	T3/3 months post- follow-up (mean, SD)	Cohen’s d (effect size)			
					Between-group d		Within-group d	
					Pre-post	Pre-FU	Pre-post	Pre-FU
Perceived stress (PSS-10)	Mindfulness *N* = 30	18.46 (5.91)	16.56 (5.30)	15.20 (6.71)	−0.11	−0.23	−0.32	−0.55
	Heartfulness *N* = 31	16.87 (6.03)	15.61 (6.10)	15.00 (5.96)			−0.21	−0.31
Depression (PHQ-9)	Mindfulness	5.26 (4.51)	4.20 (3.92)	4.80 (4.82)	−0.04	−0.03	−0.24	−0.10
	Heartfulness	4.35 (3.97)	3.48 (2.87)	4.03 (3.27)			−0.22	−0.08
Anxiety (GAD-7)	Mindfulness	6.40 (4.44)	4.63 (3.69)	5.40 (4.87)	−0.28	−0.27	−0.40	−0.23
	Heartfulness	4.53 (4.62)	4.03 (3.85)	4.74 (4.90)			−0.11	0.05
Wellbeing (WHO-5)	Mindfulness	53.46 (20.10)	58.66 (22.75)	58.80 (23.18)	−0.03	0.08	0.26	0.27
	Heartfulness	59.35 (14.42)	65.03 (16.45)	63.22 (21.42)			0.39	0.27
Wellbeing (SWEMWBS)	Mindfulness	22.23 (3.52)	22.89 (3.22)	23.58 (4.63)	0.07	0.10	0.19	0.38
	Heartfulness	23.33 (3.33)	23.75 (4.09)	24.34 (4.27)			0.13	0.30
Wellbeing (SHS)	Mindfulness	4.71 (1.31)	4.87 (1.26)	4.93 (1.30)	−0.03	−0.04	0.12	0.17
	Heartfulness	5.09 (1.30)	5.29 (1.31)	5.36 (1.16)			0.15	0.21
**Per-protocol analysis**								
**Outcome**	**Treatment group**	**Time 1/baseline (mean, SD)**	**Time 2/8 weeks Post-intervention (mean, SD)**	**Time 3/3 months post-follow-up (mean, SD)**				
Perceived stress (PSS-10)	Mindfulness *N* = 18	18.55 (6.22)	15.33 (4.28)	13.05 (6.26)				
	Heartfulness *N* = 21	15.28 (5.29)	14.57 (6.45)	13.66 (6.07)				
Depression (PHQ-9)	Mindfulness	5.22 (4.58)	3.83 (3.68)	4.83 (5.22)				
	Heartfulness	4.61 (4.53)	3.38 (2.72)	4.19 (3.32)				
Anxiety (GAD-7)	Mindfulness	6.72 (4.70)	3.55 (2.74)	4.83 (5.20)				
	Heartfulness	4.02 (4.17)	3.19 (2.54)	4.23 (4.65)				
Wellbeing (WHO-5)	Mindfulness	58.44 (19.65)	68.22 (16.26)	68.44 (17.13)				
	Heartfulness	60.76 (15.31)	69.33 (15.24)	66.66 (23.16)				
Wellbeing (SWEMWBS)	Mindfulness	22.91 (3.95)	24.10 (2.91)	25.26 (4.89)				
	Heartfulness	23.98 (3.63)	24.31 (4.63)	25.18 (4.74)				
Wellbeing (SHS)	Mindfulness	4.98 (1.19)	5.22 (1.15)	5.31 (1.19)				
	Heartfulness	5.23 (1.39)	5.39 (1.53)	5.50 (1.33)				

M, mean; SD, standard deviation. PSS-10, Perceived Stress Scvale-10; PHQ-9, Patient Health Questionnaire-9; GAD-7, Generalized Anxiety Disorder-7; WHO-5, The World Health Organization-Five Wellbeing Index; SWEMWBS, Short Warwick-Edinburgh Mental Wellbeing Scale; SHS, Subjective Happiness Scale.

Consistent with hypotheses, these findings provided no indication of differential change between interventions and comparable effectiveness across conditions. Overall, the main findings revealed that both MBI and HBI were associated with significant improvements in wellbeing and DAS symptoms over time at post-intervention (8 week) through ITT (see Table 3 below) in a total of four (PSS-10, PHQ-9, GAD-7 and WHO-5) of the six scales used in the intervention. At T3, effects were maintained for wellbeing and stress through ITT. Note that the significant reduction in anxiety (GAD-7 scores) was maintained post-follow-up only through PP analysis. Table 3 simplifies the main results for all the scales. Supplementary Mixed Models analysis sustained similar significant findings reinforcing a robust set of results.

**TABLE 3:** Test results.

		Pre-post (*F* ratio, *p*-value)			Follow-up (*F* ratio, *p*-value)		
Outcome measure	Effect	RM ANOVA - PP	Mixed model	ITT (LOCF)	RM ANOVA - PP	Mixed model	ITT (LOCF)
Stress (PSS-10)	Group	*F* = 0.70, *p* = 0.40	F = 0.80, *p* = 0.37	*F* = 0.99, *p* = 0.32	*F* = 0.57, *p* = 0.45	*F* = 1.92, *p* = 0.17	*F* = 0.51, *p* = 0.47
	Time	***F* = 4.20, *p* = 0.045**	***F* = 5.05, *p* = 0.028**	***F* = 4.12, *p* = 0.047**	***F* = 6.84, *p* = 0.002**	***F* = 14.43, *p*** ≤** 0.001**	***F* = 6.12, *p* = 0.003**
	Group × time	*F* = 0.23, *p* = 0.62	*F* = 0.28, *p* = 0.59	*F* = 0.17, *p* = 0.68	*F* = 2.08, *p* = 0.13	*F* = 1.52, *p* = 0.22	*F* = 0.44, *p* = 0.64
Depression (PHQ-9)	Group	*F* = 1.17, *p* = 0.28	*F* = 0.40, *p* = 0.52	*F* = 0.81, *p* = 0.37	*F* = 0.26, *p* = 0.61	*F* = 0.44, *p* = 0.50	*F* = 0.83, *p* = 0.36
	Time	***F* = 5.73, *p* = 0.020**	***F* = 5.24, *p* = 0.026**	***F* = 5.65, *p* = 0.021**	*F* = 2.46, *p* = 0.09	*F* = 0.47, *p* = 0.49	*F* = 2.44, *p* = 0.09
	Group × time	*F* = 0.10, *p* = 0.74	*F* = 0.04, *p* = 0.84	*F* = 0.05, *p* = 0.81	*F* = 0.01, *p* = 0.98	*F* = .017, *p* = 0.89	*F* = 0.02, *p* = 0.97
Anxiety (GAD-7)	Group	*F* = 2.14, *p* = 0.14	*F* = 1.85, *p* = 0.17	*F* = 1.78, *p* = 0.18	*F* = 1.40, *p* = 0.24	*F* = 2.04, *p* = 0.15	*F* = 1.22, *p* = 0.27
	Time	***F* = 5.23, *p* = 0.026**	***F* = 5.09, *p* = 0.028**	***F* = 4.44, *p* = 0.039**	***F* = 3.93, *p* = 0.024**	*F* = 0.98, *p* = 0.32	*F* = 2.20, *p* = 0.11
	Group × time	*F* = 1.20, *p* = 0.27	*F* = 0.76, *p* = 0.38	*F* = 1.38, *p* = 0.24	*F* = 1.61, *p* = 0.20	*F* = 0.62, *p* = 0.43	*F* = 0.85, *p* = 0.42
Wellbeing (WHO-5)	Group	*F* = 2.27, *p* = 0.13	*F* = 0.52, *p* = 0.47	*F* = 2.13, *p* = 0.14	*F* = 0.01, *p* = 0.90	*F* = 1.91, *p* = 0.17	*F* = 1.58, *p* = 0.21
	Time	***F* = 5.61, *p* = 0.021**	***F* = 5.59, *p* = 0.021**	***F* = 5.60, i = 0.021**	***F* = 6.29, *p* = 0.003**	***F* = 5.92, *p* = 0.019**	***F* = 3.45, *p* = 0.035**
	Group × time	*F* = 0.00, *p* = 0.98	*F* = 0.00, *p* = 0.93	*F* = 0.01, *p* = 0.91	*F* = 0.28, *p* = 0.75	*F* = 0.31, *p* = 0.57	*F* = 0.10, *p* = 0.90
Wellbeing (SWEMWBS)	Group	*F* = 1.08, *p* = 0.30	F = 0.97, *p* = 0.32	F = 1.45, *p* = 0.23	*F* = 0.12. *p* = 0.72	F = 1.38, *p* = 0.24	F = 1.13, *p* = 0.29
	Time	F = 1.75, *p* = 0.19	i = 1.92, *p* = 0.17	*F* = 1.72, *p* = 0.19	***F* = 3.65, *p* = 0.031**	***F* = 6.46, *p* = 0.014**	***F* = 3.48, *p* = 0.034**
	Group × time	*F* = 0.10, *p* = 0.7	*F* = 0.13, *p* = 0.71	*F* = 0.08, *p* = 0.77	*F* = 0.41, *p* = 0.66	*F* = 0.30, *p* = 0.58	*F* = 0.07, *p* = 0.92
Wellbeing (SHS)	Group	*F* = 1.6, *p* = 0.21	*F* = 0.51, *p* = 0.47	*F* = 1.64, *p* = 0.20	*F* = 0.27, *p* = 0.60	*F* = 0.96, *p* = 0.33	*F* = 1.86, *p* = 0.17
	time	*F* = 2.12, *p* = 0.15	*F* = 2.29, *p* = 0.13	*F* = 2.14, *p* = 0.14	*F* = 2.20, *p* = 0.11	***F* = 5.00, *p* = 0.030**	*F* = 2.33, *p* = 0.10
	Group × time	*F* = 0.00, *p* = 0.92	*F* = 0.01, *p* = 0.90	*F* = 0.02, *p* = 0.88	*F* = 0.05, *p* = 0.94	*F* = 0.00, *p* = 0.92	*F* = 0.02, *p* = 0.97

This table shows the significant results of the study in bold. *P <0 .05.

### 3.3 Within-group changes in MBI and HBI—secondary outcomes

The within-group analyses were performed to assess pre-post changes in DAS symptoms and wellbeing within each of the two evaluated interventions. Both MBI and HBI were associated with improvements over time, including reductions in DAS symptoms and increasing wellbeing levels, with effect sizes ranging from small to moderate (d = 0.12 to 0.55). The moderate improvement was observed in stress for the MBI.

Pre-test (T1), post-test (T2), and post-follow-up test (T3) mean values and standard deviations of stress, anxiety, depression and wellbeing in MBI and HBI groups. Cohen’s d: Small effect at <0.5, Medium effect at 0.5, Large effect at >0.8.

Mauchly’s test of sphericity indicated that the assumption of sphericity was violated in all the RM ANOVA outputs for the main effect of Time, W (1) = 1,000, *p* = 0.045 (E.g.: PSS-7 scale). However, Greenhouse-Geisser correction was automatically applied helping to maintain power while controlling for type I errors by adjusting degrees of freedom (Field, 2018).

#### 3.3.1 Perceived stress

Figure 2A shows that the RM ANOVA results revealed significant main effect over time (within groups) in reducing the identified outcome variable perceived stress indicating a positive change in scores from pre- to post-intervention and also post-follow-up as showed in Table 3 [*F*_(1, 55)_ = 6.12, *p* = 0.003] for both HBI and MBI cohorts through ITT analysis.

**FIGURE 2:**
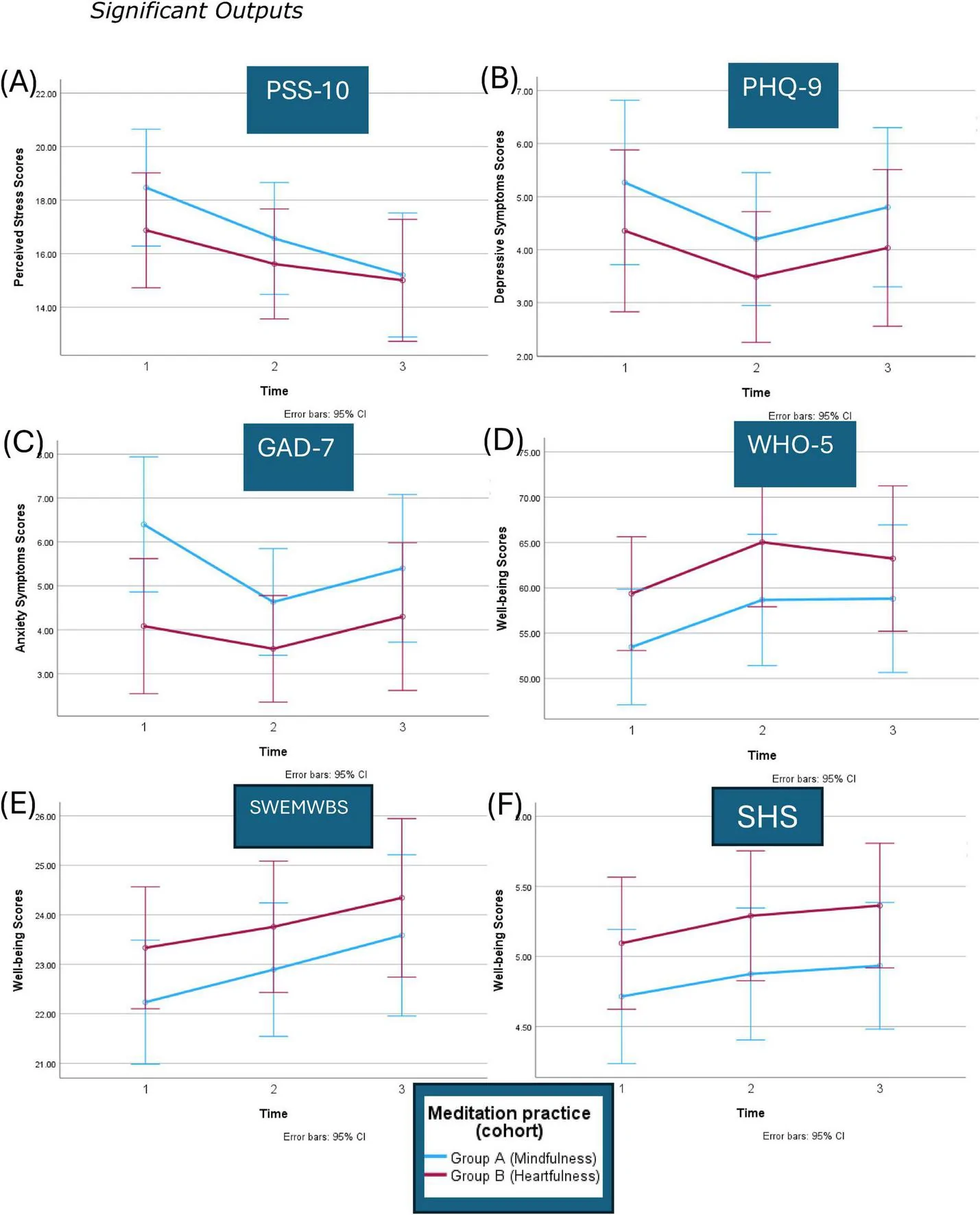
Significant outputs. Line graphs show the effects of time on panel **(A)** stress (PSS-10), **(B)** depression symptoms (PHQ-9), **(C)** anxiety (GAD-7), **(D)** well-being (WHO-5), **(E)** wellbeing (SWEMWBS) and **(F)** subjective happiness (a key component of wellbeing) (SHS). Through ITT analysis, significant reductions in stress, depression symptoms, and anxiety, alongside an increase in wellbeing (WHO-5) were observed following the 8-weeks intervention (T1-T2) in both the Mindfulness and Heartfulness groups. At the 12-weeks follow-up (T1-T3), the significant effect of time was maintained for stress (reduction) and wellbeing (WHO-5) (increase) in both groups through ITT analysis. Anxiety (GAD-7) continued to decrease at follow-up only through PP analysis. SWEMWBS showed a delayed effect of time, with an increase in well-being observed only at follow-up through ITT analysis. SHS did not show a significant main effect of time.

#### 3.3.2 Depression

Figure 2B results indicated an initial significant main effect over time (within groups) in reducing the outcome variable depressive symptoms (*F*_(1, 55)_ = 5.65, *p* = 0.021) from T1 to T2 for both HBI and MBI cohorts through ITT analysis. However, it was not maintained post follow-up sessions.

#### 3.3.3 Anxiety

Figure 2C indicates that GAD’s also presented an initial main significant effect over time (within groups) for both Heartfulness and Mindfulness cohorts in reducing the outcome variable anxiety [*F*_(1, 55)_ = 4.44, *p* = 0.039]. However, it was maintained post-follow-up only through the Per-Protocol (PP) RM ANOVA results [*F*_(1,55)_ = 3.93, p = 0.024]. It was not sustained post-follow-up through ITT analysis.

#### 3.3.4 Wellbeing

Figure 2D highlights the estimated marginal means changes of wellbeing levels in the WHO-5 WBI scale. It was found that wellbeing levels increased significantly over time (within groups) for both HBI and the MBI from T1 to T2 through ITT analysis. The significant effect was maintained post-follow-up sessions after 3 months [F_(1, 55)_ = 3.45, *p* = 0.035].

Figure 2E displays that the SWEMWBS scale did not reveal any initial significant main effect over time (within groups) for both HBI and MBI groups from T1 to T2. However, a delayed effect of time was presented after follow-up sessions at T3 indicating the T1 to T3 effect was significant [*F*_(1, 55)_ = 3.48, *p* = 0.034].

Figure 2F shows that the SHS scale did not present any significant main effect over time (within groups) for both HBI and MBI groups [*F*_(1, 55)_ = 2.16, *p* = 0.14]. However, the descriptive statistics Table 2 indicate that the outcome variable wellbeing also increased for both groups after intervention completion.

## 4 Discussion

In line with the hypothesis of this study, our primary outcomes focused on between-group differences and demonstrated comparable effects through an initially powered RCT design. These findings may be particularly valuable in learning more about the relative impact of HBIs where evidence is more limited. Secondary within-group outcomes demonstrated positive outcomes across intervention arms. Both MBI and HBI produced small but statistically significant improvements on stress, anxiety, depressive symptoms and wellbeing at T2 post-8-week-interventions. Improvements were sustained for stress and wellbeing thought ITT analysis at T3 post-twelve-week-follow-up (spanning 5 months overall), while anxiety improvements were only maintained through PP analysis. This suggests the study was able to detect meaningful within-group changes. A moderate effect size was also registered in enhancing stress for the MBI group. However, these findings should be interpreted cautiously given the exploratory nature of this RCT. They may help inform future larger definitive trials to validate the effects of MBI and HBIs, particularly in studies using similar outcome assessments. These findings emphasize the potential limits of the 8-week (1 h each) MBI and HBI in terms of an enduring impact without more regular contact with the practices, since the 12-week follow-up sessions occurred only once a month. The short-term benefit of these interventions, however, goes beyond prevention, reducing risk factors of mental ill-health when experiencing loneliness and DAS. They have therefore conferred potential trans-therapeutic benefits among distance postgraduate students, who expressed overall enhancements in their mental health and wellbeing. These findings are in accordance with previous literature supporting the positive impact of both Mindfulness and Heartfulness on psychological and emotional wellness on a wide range of individuals including university students, healthcare professionals, outpatients with diabetes, women with recurrent pregnancy loss, and post-COVID-19 patients with increased stress and impaired sleep quality (Amarnath et al., 2018; Thimmapuram et al., 2017, Tovote et al., 2014; Jensen et al., 2021; Subramanian et al., 2022; Desai et al., 2021).

In addition, a limitation within the existing literature is the absence of studies employing active comparator conditions in Mindfulness-based intervention research, as many studies have relied on passive controls such as waitlist or usual-care groups. This limitation is even more substantial in the Heartfulness meditation literature, where comparative intervention trials are currently lacking. The present study is the first to directly compare a Heartfulness-based intervention with another active intervention condition. Consequently, these findings contribute to the growing comparative literature on MBIs while also providing novel evidence regarding the comparative benefits of Heartfulness meditation, helping to address an important methodological gap in the scientific literature. Future research involving larger definitive RCTs with active comparator groups may be required to further establish the relative efficacy of HBIs and MBIs.

Overall, attrition was considered substantial (40%), but dropout was comparable between groups after follow-up (3.7%), suggesting attrition would not introduce differential bias. Improvements in mental health outcomes remained consistent despite participant loss through the sensitivity analyses, RM ANOVA and mixed-modeling findings. Stability of scores was assumed over time using LOCF for missing data. However, true changes in mental health outcomes may be underestimated with inclusion of all randomized participants to acknowledge potential bias. PP analysis was also conducted with participants that completed the interventions, excluding dropouts and showing full effect. The attrition results highlight the importance of strategically formulating a way of maintaining students in interventions like this. The study has nevertheless shown a positive impact on participant retention since researchers expected 25% loss after the 8-week-sessions (with 40 participants targeted in each group) and registered only 12.3% loss (with 31 and 34 participants in each group), which can be considered a low attrition rate.

There may be a protective mental health impact or increase in resilience stemming from the enhancement of mental health and wellbeing that students experienced from the practices. For example, in supporting them to potentially face challenging life events differently, without experiencing the same degree of stressful reactions to reinforce overall positive wellbeing effects.

While acknowledging the distinction in approach between MBI and HBI, the results of this study nevertheless imply that both of these approaches are observed as effective. Both could be offered as evidence-based treatment for mental health support to enhance wellbeing and reduce stress, anxiety and depressive symptoms. These benefits are potentially also feasible to achieve with other distance-learning postgraduate student groups, university students, and perhaps final-year high school students. The interventions could also be beneficial among individuals and teams working/living remotely, during lockdowns, or during post-pandemic recovery.

This study suggests that while the evidence base is still less well developed, Heartfulness meditation programmes may be considered as an alternative, or comparable approach to Mindfulness-based programmes in supporting the enhancement of mental-wellbeing. This is a novel finding and indicates Heartfulness may represent an alternative approach to supporting mental health in a range of contexts. It also invites the opportunity of conducting further confirmatory larger studies to better understand the underlying mechanisms it draws on to enhance mental health outcomes. The brief MBI of the current study may also be considered a novel approach for mental wellness purposes because it was delivered for 1 h per week, rather than the standard weekly sessions lasting 2–2.5 h across 8 weeks.

### 4.1 Limitations and strengths

Although findings are promising, a number of limitations should be taken into account. Despite the achieved sample size remaining comparable to sample sizes commonly reported in MBI trials and related meta-analytic literature (Hofmann et al., 2010), it was still slightly lower than the a priori target. Consequently, this study may have had slightly reduced sensitivity to detect moderate between-group effects. The primary outcomes focused on between-group differences, consistent with the RCT design according to the hypothesis proposed. The indication of comparable effects between the MBI and HBI finding may be particularly valuable in learning more about the relative impact of HBIs where evidence is more limited. Secondary within-group outcomes demonstrated positive findings across intervention arms. Both MBI and HBI produced small but statistically significant improvements on stress, anxiety, depressive symptoms and wellbeing, suggesting the study was able to detect meaningful within-group changes. A moderate effect size was also registered in enhancing stress for the MBI group.

The absence of significant between-group differences should nonetheless be interpreted cautiously, as the study may have been underpowered to detect moderate effects. As an exploratory initially powered RCT, the findings should be viewed as preliminary evidence consistent with comparable effects across MBI and HBI. Further tests developed through a fully powered RCT may be required to fully assess equivalence. This RCT may therefore help future definitive trials to validate the effects of either an MBI or HBI, particularly in studies using similar outcome assessments.

While research studies focusing exclusively on the mental health of distance learning postgraduate students remain under-explored, this study is pioneering in offering illuminating data about levels of stress, anxiety, depressive symptoms and wellbeing in these students through well-established, validated measures. Further research may now be required to investigate potential challenges faced by distance postgraduate students that could lead to lower levels of wellbeing and increased risks of poor mental health. It should also be replicated to ensure greater robustness and generalizability of findings.

Another limitation is that there are some disadvantages linked with psychometric self-report measures including participants being biased when reporting their own feelings and experiences such as underreporting psychological distress due to stigma, judgment, confidentiality concerns or even lack of self-awareness, because they fear demonstrating they are mentally unwell (Sunderland et al., 2019). On the other hand, these scales can have many advantages such as practicality, low cost and time efficiency to be administrated and interpreted assessing common mental disorders’ classifications and status, while conferring reliability in reporting as validated measures (Sunderland et al., 2019).

The ITT analysis conducted in this study was compared with the PP outputs to obtain a more complete scenario of the treatment effects, reinforcing the reliability of the study (Ahn and Kang, 2023). However, there is some discussion in the scientific field around the primary interest of trials being focused on the effectiveness of treatments (Keene et al., 2021; Silverman et al., 2024). This may be particularly relevant in a head-to-head study where the effectiveness of each intervention is the focal point, which suggests PP analysis should be given greater analytical value in this case. Although this study mainly focuses on ITT analysis as the gold standard approach, PP approach outputs are also published in Table 3 showing the significant results in bold. Further investigation about the importance of PP analysis for these types of studies are needed to clarity its importance. This study did not measure potential process variables (mechanism of change of the intervention) such as trait mindfulness, mindfulness, psychological flexibility and others.

Due to the high occurrence and sustained impact of psychological challenges arising from managing academic pressures and overwhelm linked to personal and professional issues, or difficulties triggered by uncertain times, a strength of this study is its global reach among postgraduate participants from different parts of the world. This study showed that MBI and HBI may be low-cost approaches and efficient over a short period, especially remotely. Implementing mental health preventative measures through wellbeing interventions that target distance postgraduate students may offer more supportive effects than reactive strategies for this academic group that often experiences high levels of stress, anxiety and depression compared to the overall population. However, future research with this population is required (Chung et al., 2021), especially in larger and more diverse distance postgraduate students’ groups.

### 4.2 Future directions

As this was a head-to-head study comparing one active treatment to another, novel investigations could evaluate effects in a no-treatment group with either MBI or HBI on distance-learning post-graduate students. Although studies have shown that improvements in mental health can influence in better academic or scholar achievement (Song and Hu, 2024; Medlicott et al., 2021; Voltmer et al., 2023), this was not investigated in this research since it was not part of the ethical agreement with participants. These interventions (MBI or HBI), however, may be also framed as supportive for students’ educational progress. Future research should also explore if the synchronous remote/online programmes could positively influence intervention results alone, by their potential to increase interaction and sense of community among participants (Oztok et al., 2013). This study also proposes to explore the potential for these alternative programmes to be offered as additional mental health support, since universities tend to only offer individual online counseling sessions if requested by students. In addition, a future larger definitive trial may consider the follow-up outcome (12-weeks or a later long-term assessment) as the most appropriate primary endpoint. Future studies should also examine whether maintaining a similar or higher intervention frequency during follow-up would improve durability of effects.

## 5 Conclusion

This is the first head-to-head RCT investigating the comparative effects of a HBI and MBI in reducing perceived stress, anxiety and depressive symptoms, and in increasing wellbeing among distance master’s students. These findings are consistent with comparable effects, and the interventions may be considered as beneficial preventative and transdiagnostic approaches for postgraduate students‘ mental health and wellbeing. Even though the study was not fully powered as a definitive equivalence trial, these results clearly suggest that both HBI and MBI are promoting similar effects on students’ mental wellbeing. This research reinforces the imperative for further strategies to enhance the mental health of students through reliable evidence and regular wellness programmes in universities.

Approaches such as HBI and MBI, based on meditation, may be feasible, cost-effective and efficient as evidenced in this study. Ultimately, our findings are consistent with effects being comparable, with further larger scale fully powered studies required to confirm this. Future research is warranted to replicate and extend these findings among master’s and potentially doctorate students; cohorts with clearly evidenced need for wider mental health support. Lastly, given the effectiveness observed, this study provides promising initial evidence suggesting a brief eight-week remote/online synchronous guided Heartfulness or Mindfulness meditation programme could be used as additional preventative wellbeing approaches to the traditional university system (e.g., counseling). In particular, to support postgraduate students, including those experiencing mental health crisis, at scale and remotely with a global reach.

## Data Availability

The raw data supporting the conclusions of this article will be made available by the authors, without undue reservation.
